# Supplementary material for: The acoustical behavior of a bass guitar bridge with no saddles
Source: PLoS One. 2023 Oct 25;18(10):e0292515. doi: 10.1371/journal.pone.0292515 (PMC10599535; doi:10.1371/journal.pone.0292515)
Supplement: Attachment — Submitted filename: Response to Reviewers.pdf [file pone.0292515.s001.pdf]

University of St Andrews
Scotland's first university

600 YEARS
1413 – 2013

Department of Music
Jonathan A. Kemp BSc, PhD, LLCM(TD) MIOA
Teaching Fellow

Dr. Jonathan A. Kemp
Laidlaw Music Centre,
Queen's Gardens,
University of St Andrews,
St Andrews,
Fife,
KY16 9QF
+44 1334 46 2145
jk50@st-andrews.ac.uk

Dear Sir/Madam,

Thank-you for the review of my paper "The acoustical behavior of a bass guitar bridge with no saddles". I am grateful for the feedback and believe that responding to these has enhanced the paper. I have used latexdiff to track changes on the manuscript. Here are my responses to editor and reviewer points:

1. Please ensure that your manuscript meets PLOS ONE's style requirements, including those for file naming. The PLOS ONE style templates can be found at
https://journals.plos.org/plosone/s/file?id=wjVg/PLOSOne_formatting_sample_main_body.pdf and
https://journals.plos.org/plosone/s/file?id=ba62/PLOSOne_formatting_sample_title_authors_affiliations.pdf

I believe I have done this.

2. Please note that PLOS ONE has specific guidelines on code sharing for submissions in which author-generated code underpins the findings in the manuscript. In these cases, all author-generated code must be made available without restrictions upon publication of the work. Please review our guidelines at https://journals.plos.org/plosone/s/materials-and-software-sharing#loc-sharing-code and ensure that your code is shared in a way that follows best practice and facilitates reproducibility and reuse.

I have now uploaded data to a public Open Science Framework repository called "The acoustical behavior of a bass guitar bridge with no saddles" with identifier:
DOI 10.17605/OSF.IO/TBZDC
at https://osf.io/tbzdc/

3. Thank you for stating the following financial disclosure:
"No funding was applied. All items used in the study were purchased by the author, or provided free in the case of the Ray Ross bridge, or was equipment owned by the University of St Andrews."

At this time, please address the following queries:

a) Please clarify the sources of funding (financial or material support) for your study. List the grants or organizations that supported your study, including funding received from your institution.

The University of St Andrews is a charity registered in Scotland, No: SC013532

b) State what role the funders took in the study. If the funders had no role in your study, please state: "The funders had no role in study design, data collection and analysis, decision to publish, or preparation of the manuscript."

c) If any authors received a salary from any of your funders, please state which authors and which funders.

d) If you did not receive any funding for this study, please state: "The authors received no specific funding for this work."

Please include your amended statements within your cover letter; we will change the online submission form on your behalf.

I have included "The authors received no specific funding for this work" in the Acknowledgements section of the manuscript.

4. We note that you have indicated that data from this study are available upon request. PLOS only allows data to be available upon request if there are legal or ethical restrictions on sharing data publicly. For more information on unacceptable data access restrictions, please see http://journals.plos.org/plosone/s/data-availability#loc-unacceptable-data-access-restrictions.

In your revised cover letter, please address the following prompts:

a) If there are ethical or legal restrictions on sharing a de-identified data set, please explain them in detail (e.g., data contain potentially sensitive information, data are owned by a third-party organization, etc.) and who has imposed them (e.g., an ethics committee). Please also provide contact information for a data access committee, ethics committee, or other institutional body to which data requests may be sent.

b) If there are no restrictions, please upload the minimal anonymized data set necessary to replicate your study findings as either Supporting Information files or to a stable, public repository and provide us with the relevant URLs, DOIs, or accession numbers. For a list of acceptable repositories, please see http://journals.plos.org/plosone/s/data-availability#loc-recommended-repositories.

We will update your Data Availability statement on your behalf to reflect the information you provide.

There are no ethical or legal restrictions on the data. I have now uploaded data to a public Open Science Framework repository called "The acoustical behavior of a bass guitar bridge with no saddles" with identifier: DOI 10.17605/OSF.IO/TBZDC at https://osf.io/tbzdc/

Reviewers' comments:

Reviewer's Responses to Questions

Comments to the Author

1. Is the manuscript technically sound, and do the data support the conclusions?

The manuscript must describe a technically sound piece of scientific research with data that supports the conclusions. Experiments must have been conducted rigorously, with appropriate controls, replication, and sample sizes. The conclusions must be drawn appropriately based on the data presented.

Reviewer #1: Yes

Reviewer #2: Partly

2. Has the statistical analysis been performed appropriately and rigorously?

Reviewer #1: N/A

Reviewer #2: No

3. Have the authors made all data underlying the findings in their manuscript fully available?

The PLOS Data policy requires authors to make all data underlying the findings described in their manuscript fully available without restriction, with rare exception (please refer to the Data Availability Statement in the manuscript PDF file). The data should be provided as part of the manuscript or its supporting information, or deposited to a public repository. For example, in addition to summary statistics, the data points behind means, medians and variance measures should be available. If there are restrictions on publicly sharing data—e.g. participant privacy or use of data from a third party—those must be specified.

Reviewer #1: No

Reviewer #2: Yes

4. Is the manuscript presented in an intelligible fashion and written in standard English?

PLOS ONE does not copyedit accepted manuscripts, so the language in submitted articles must be clear, correct, and unambiguous. Any typographical or grammatical errors should be corrected at revision, so please note any specific errors here.

Reviewer #1: Yes

Reviewer #2: Yes

5. Review Comments to the Author

Please use the space provided to explain your answers to the questions above. You may also include additional comments for the author, including concerns about dual publication, research ethics, or publication ethics. (Please upload your review as an attachment if it exceeds 20,000 characters)

Reviewer #1: This is a clear account of a well-defined question, and I only have very minor comments.

First, the author has surely not complied with the instructions about data availability? You specifically forbid "Available from the author"

I have now uploaded data to a public Open Science Framework repository called "The acoustical behavior of a bass guitar bridge with no saddles" with identifier: DOI 10.17605/OSF.IO/TBZDC at at https://osf.io/tbzdc/

Throughout, we have an odd mixture of inches and mm as units. I understand that strings may be marketed in inches, but for a scientific publication it would surely be better to use SIU throughout?

Guitar strings and winding materials are sold and manufactured in imperial units for diameter, hence the choice has been made to use imperial units for these parameters. Metric units are used everywhere else. This is consistent with my previous work published in PLOS One and SN Applied Sciences so I would prefer not the change all units to SIU.

In the discussion on the Grand Piano, it seems very odd not to reference the famous paper by Weinreich.

This paper has now been referred to in the "Introduction" section and the text amended to account for the importance of tuning differences for controlling decay rate.

Finally, a few typos:
Line 55 "with first with"
Line 211 "sting"
Line 239 "inharomonicty"
Line 321 "deigns"

These typos have now been corrected thanks.

Reviewer #2: Review: PONE-D-23-19708
Title: The acoustical behavior of a bass guitar bridge with no saddles

The paper deals with the comparative acoustic analysis of bass guitar strings depending on the type of bridge used (conventional, respectively Ray Ross bridge). The article is interesting both theoretically and experimentally. However, there are some aspects that should be improved so that it is understood.

The first aspect that I recommend is related to the organization of the chapters, as there should be a section in which the two methods addressed (experimental and analytical modelling) are presented, perhaps even the author will introduce a logical scheme of the research concept presented in this article.

Thus, the methods used in this study are: analytical, experimental and data processing.

The experimental ones aim at three components: testing the G string with two types of bridges, testing in a musical context (comparisons between all strings) and finally, testing the piano string, respectively electronic chorus effect.

The theoretical chapter, Theory for the effect of the knot, could be moved higher, after the Introduction section.

The theory section has been moved to be before the experiments.

I have also added further clarification to the reasons for the different methods employed. For instance in the Introduction: "Since theoretical considerations predict that the inharmonicity is reduced to the greatest extent for the thinnest (G2) string on the bass guitar, the experiments involve sensitive measurements using an optical pickup of the correct size for that string to validate the theory. In order to ensure generality of results when considering sustain in realistic musical context, all four strings from a standard set of strings from a different manufacturer were measured in the later experiments with magnetic pickups installed."

Introduction

In the explanations given in the Introduction, paragraphs 19 - 28, a figure should be added to exemplify the two ways of holding the strings on the bridge.

The photos of the different bridges with strings installed are given in the subsequent section and I feel this is clear enough in terms of illustrating how the strings are held on the instrument for the two designs.

Experimental comparison of bridge designs for G string using optical pickups

In the description of the experiment, can you indicate at what distance from the bridge the optical pickup was placed?

This has now been set out, along with the implications regarding measuring at or close to nodes.

Why did you choose to analyse the G string and not the others?

This has now been discussed. The G string has more inharmonicity reduction so is good choice for detailed testing using optical pickups (which have to be sized and positioned precisely for good measurements) in order to validate the theory. The size of inharmonicity reduction expected depends on the string construction but typical relative strength of inharmonicity reduction for thicker strings is now discussed at the end of the theory section. All strings were measured for sustain.

In the section Experimental comparison of bridge designs sustain for all strings, you mentioned that the comparative acoustic analysis of the 4 strings was done with D'Addario EXL170 strings, unlike the first stage of the experiment in which the G string was made by Sadowsky Blue. Why didn't you use the same type of strings throughout your studies? What implications does the type of string have on inharmonicity and sustain?

The type of roundwound string does not have major implications for sustain, particularly for relative comparison of strings on the different bridges. I have added this.

Both in the section and Experimental comparison of bridge designs for G string using optical pickups and In the section Experimental comparison of bridge designs sustain for all strings, the graphs with the time analysis of the damped signal of the tested strings should be added.

The acoustics package used did not give graphs of the time analysis, but rather gives values based on internal filtering and internal time decay analysis. This package was used to help with reproducibility. I feel the paper would contain too many pages time decays graphs were added (especially since each data point is based on a different band pass filtered signal). I could add the non-filtered decays (which of course are not close to straight lines of dB against time) or an example of a filtered decay if still requested.

Finally, I recommend the major revision of the paper so that it can be published.

6. PLOS authors have the option to publish the peer review history of their article (what does this mean?). If published, this will include your full peer review and any attached files.

If you choose "no", your identity will remain anonymous but your review may still be made public.

Do you want your identity to be public for this peer review? For information about this choice, including consent withdrawal, please see our Privacy Policy.

Reviewer #1: No

Reviewer #2: No

Thanks again for the excellent points regarding reviewing the article. Do not hesitate to request additional changes if required.

Yours, Jonathan Kemp
